# Unmasking the effects of E-leadership on virtual team effectiveness by an integrated fsQCA and NCA method

**DOI:** 10.1371/journal.pone.0331500

**Published:** 2025-09-12

**Authors:** Yining He, Cong Cheng, Limin Wang

**Affiliations:** 1 School of Economics and Management, Zhejiang Shuren University, Hangzhou, Zhejiang Province, China; 2 School of Management, Zhejiang University of Technology, Hangzhou, Zhejiang Province, China; 3 Department of Accountancy, Zhejiang Gongshang University Hangzhou College of Commerce, Hangzhou, Zhejiang Province, China; Universiti Tenaga Nasional, MALAYSIA

## Abstract

Together with unstoppable trend of business going digitally global in recent years and the worldwide spread-out of COVID-19 since 2020, virtual teams have gradually garnered considerable attention in academia. Yet, the question of how to boost virtual team effectiveness remains underexplored. This study adopts a leadership perspective to examine the role of E-leadership in enhancing virtual team effectiveness. Gathered survey data from 74 virtual teams of Chinese manufacturing firms and employed an integrated fsQCA and NCA method, this study unravels two primary results. First, Fuzzy-set Qualitative Comparative Analysis (fsQCA) identified the sufficient combinations of E-leadership dimensions to promote virtual team effectiveness. Second, Necessary Condition Analysis (NCA) specifies the quantitative thresholds a E-leadership dimension’s value must be to render different levels of virtual team effectiveness. The conclusions of this research offer valuable insights into the theoretical and managerial implications of E-leadership in virtual teams.

## Introduction

In today’s highly competitive global economy, businesses are increasingly relying on virtual teams to meet the demands of geographically distributed markets, a trend accelerated by the COVID-19 pandemic. Virtual teams, characterized by their reliance on information and communication technology (ICT) and their operation across spatial and temporal boundaries, offer significant advantages such as flexibility, scalability, and access to diverse talent [1]. However, these teams also face a set of challenges that impact their effectiveness, including communication breakdowns, trust deficits, and difficulties in technology adoption [2].

Effective leadership is crucial in overcoming these challenges and optimizing the performance of virtual teams. While traditional leadership models such as transformational leadership [3] and shared leadership [4] have been applied to virtual teams, they often fall short in addressing the complexities introduced by the interplay between human dynamics and ICT. To bridge this gap, Avolio et al. (2000) introduced E-leadership, emphasizing the integration of leadership practices with ICT to enhance team effectiveness [5]. E-leadership has received broad recognition thereafter, with its implications and characteristics further expanded [6–8]. E-leadership not only facilitates communication and coordination but also focuses on managing the human-technology interface, which is crucial for virtual team success.

Although the growing importance of E-leadership has been widely recognized, many existing studies examine specific leadership behaviors such as task coordination, motivation, or communication in isolation and inconsistency exists as to the conclusions of the literatures [7–9]. For example, Jarvenpaa & Leidner (1999) confirm that early-stage “swift trust” in virtual teams, which is often based on initial impressions, is fragile and tends to deteriorate over time if not actively maintained. This decline can lead to reduced cohesion, collaboration, and knowledge sharing [10].While Schilke et al. (2016) found a moderate positive correlation between trust and team effectiveness, with the effect being stronger in virtual teams than in face-to-face ones [11]. Those paradox results call for a configurational theory approach which enables us to shift our attention from individual variables to holistic configuration [12–13]. This approach such as fsQCA assume that the influence of attributes (E-leadership attributes) on a specific outcome (virtual team effectiveness) depends on how the different attributes are combined, rather than on the levels of the individual attributes per se. In addition, most prior research treats virtual teams as homogeneous and static, overlooking how leadership needs may shift depending on team maturity, task complexity, or environmental volatility [14]. Addressing these two gaps, this study thus adopts a configurational and necessity-based approach to explore how multiple E-leadership dimensions work together, and what are the bottleneck attributes for further enhancing virtual team effectiveness across different contexts.

Accordingly, this study aims to fill the two gaps by investigating the following research questions. Q1: What combinations of E-leadership dimensions promote virtual team effectiveness? Q2: What are the necessary E-leadership attributes under varying degrees of virtual teams performance? To achieve these objectives, we employ an integrated methodological approach combining fuzzy-set Qualitative Comparative Analysis (fsQCA) and Necessary Condition Analysis (NCA). fsQCA is used to identify multiple combinations of E-leadership dimensions that jointly contribute to high team effectiveness, reflecting the causal complexity of leadership in virtual settings. Meanwhile, NCA helps to determine which specific E-leadership elements serve as necessary conditions, the critical thresholds that must be met for teams to perform effectively. Thus, by uncovering the configurations of E-leadership dimensions that lead to high team performance, this study layout the multiple equifinal pathways for managers to realize virtual team effectiveness. Taking a step further by identifying the bottlenecks that constrain different levels of team performance, this study pinpoints the specific leadership behaviors that managers should prioritize to improve outcomes in virtual teams.

Our findings reveal three main conclusions. First, virtual team effectiveness is not driven by any single E-leadership dimension but rather by the synergistic integration of multiple practices, such as communication, trust-building, and technology management. Second, different configurations yield equifinal leadership pathways. Third, virtual team effectiveness is contingent upon specific dimensions meeting requisite thresholds; deficiencies in these necessary conditions undermine performance, notwithstanding strengths of other attributes. These insights enrich our understanding of E-leadership by demonstrating not only what combinations of practices are effective, but also which dimensions are essential for unlocking team potential.

## Theoretical background and hypothesis development

### Virtual team

Virtual teams are characterized as groups of geographically dispersed employees. These teams, consisting diverse departments, such as R&D, customer service, and project teams, enable organizations to bridge discontinuities in time and geography, thereby leveraging scattered resources effectively. A significant focus within the research area of virtual teams pertains to their effectiveness. Virtual teams are recognized for their capacity to enhance responsiveness to market shifts, reducing management costs, facilitating continuous workflows, and securing information benefits. Nevertheless, increased levels of virtuality may adversely affect team functionality. Alsharo et al. (2017) highlight that the geographic dispersion inherent in virtual teams is often inversely related to their effectiveness [2]. Correspondingly, Presbitero (2021) notes that heightened virtuality diminish trust and coordination within teams [15]. Furthermore, the substantial cultural differences among virtual team members create disparities in information interpretation and engender defensive attitudes [16]. Such challenges impede individuals’ ability to effectively coordinate with their teammates, detrimentally impacting both team processes and outcomes. Therefore, it is imperative to delve deeper into strategies that enhance the effectiveness of virtual teams.

### From leadership to E-leadership

Earlier research in leadership primarily focused on the dynamics between leaders, members, and the context shaping their interactions [17]. However, the advent of ICT has fundamentally transformed these contexts and the nature of leader-member relationships [18]. The virtual realm has increasingly supplanted physical interactions. Consequently, leaders must navigate not just the physical presence of members but also their interplay with ICT. Traditional leadership paradigms are inadequate for addressing the demands of modern efficient, and dynamic business operations, highlighting the necessity of E-leadership in new patterns of information acquisition, storage, and interpretation within virtual environments. E-leadership is defined as a social influence process that operates within both close and distant contexts, mediated by advanced ICT, and is capable of effecting changes in attitudes, emotions, thoughts, behaviors, and performance [19]. Research indicates that E-leadership emerges from the intersection of ICT and leadership processes [5,20].

E-leadership has gained significant attention due to its crucial role in managing organizations, both in educational institutions and business organizations. In both contexts, E-leaders are tasked with leveraging digital tools to enhance communication, collaboration, and overall team effectiveness. As organizations embrace online platforms for learning or business operations, E-leadership becomes essential in managing both technological and human aspects of virtual environments. E-leaders must facilitate effective communication, engagement, and motivation among team members, using tools such as video conferencing, learning management systems, and collaborative platforms to ensure alignment with organizational goals [21].

In educational settings, E-leaders focus on fostering an interactive and inclusive online learning experience, leveraging adaptive leadership practices to enhance student engagement and academic performance, which is crucial for ensuring an inclusive and effective online environment [22]. Additionally, fostering trust and collaboration in online environments where face-to-face interaction is limited is a key challenge for E-leaders in education [23]. Similarly, in business organizations, E-leaders must address the challenges of remote work by adopting flexible leadership styles to overcome communication barriers, build trust, and manage dispersed teams effectively [19]. Regardless of the setting, E-leaders must be proficient in integrating technology with organizational processes, ensuring that digital tools enhance rather than disrupt the work experience.

A critical aspect of E-leadership is the ability to navigate technology, whether it be through learning management systems, collaborative platforms, or through business management tools, to facilitate real-time collaboration and decision-making across geographically dispersed teams [8,24]. Furthermore, E-leaders also play a key role in managing information systems, digital collaboration tools, and digital supply chains, all of which are integral to optimizing team performance in both education and business environments [14]. In essence, E-leadership combines leadership skills with technological expertise to streamline operations, improve efficiency, and ensure organizational success, making it a versatile and essential approach to managing contemporary organizations.

### E-leadership in virtual teams

E-leadership is increasingly becoming integral to the virtual world of global business [8]. It represents not just a significant adaptation to evolving business realities but also a novel approach to influence team management. Thus, the essence of E-leadership can be distilled into two key aspects. First, it involves the synergy between leadership and ICT. Effective management of E-change necessitates a fusion of leadership skills and ICT expertise. E-leaders integrate elements such as information system management, enterprise resource planning, digital supply chain management, and online customer relationship management to expedite information flow and enhance operational efficiency. Second, E-leadership entails the management of ICT utilization. E-leaders provide technical guidance to team members to foster their understanding, acceptance, and expertise in ICT. This includes alleviating apprehensions about new technologies and facilitating training to advance their technical skills, such as knowledge of technological progress and the capacity for technical support [24]. Concurrently, E-leaders are tasked with developing comprehensive technical protocols and swiftly addressing technical issues, to ensure rational and effective technology employment in virtual team operations.

Previous research has extensively examined the role of E-leadership within virtual teams, identifying various attributes of it. We consolidated these studies into Table 1 and further synthesized them into five distinct aspects. It is important to note that among these identified attributes, technical guidance forms the foundation of E-leadership and should not be considered a separate function. Moreover, effective communication serves as the cornerstone for realizing the potential of E-leadership. Consequently, we categorized the core functions of E-leadership into the following five areas: trust building, task management, well-being safeguarding, adaptation management, and innovation management.

**Table 1:** Configuration of E-leadership.

Configuration of E-leadership	Zigurs (2003) [25]	Malhotra et al. (2007) [6]	Fernandez & Jawadi (2015) [7]	Dulebohn & Hoch (2017) [9]	Roman et al. (2019) [8]	Chamakiotis et al. (2021) [26]
Technology guidance	✓				✓	
Team construction		✓	✓	✓	✓	✓
Task completion	✓	✓	✓			
Communication				✓	✓	
Work-life management		✓				✓
Innovation					✓	✓
Member		✓	✓		✓	
Trust construction					✓	

#### Trust building.

A highly digitized environment complicates trust-building within management processes, particularly in virtual teams. In non-face-to-face interactions, the absence of physical and social cues such as facial expressions and voice intonation, makes it difficult to assess emotions and intentions accurately [27]. This lack of personal connection often leads to a scarcity of informal interactions and humor, which are critical for building social bonds and mutual trust in traditional, face-to-face teams. Furthermore, virtual interactions generally occur in digital spaces, where the visibility of team members is limited, deepening the sense of isolation. This physical separation can diminish mutual commitment and hinder the development of interpersonal relationships, reducing trust among team members [28]. Another challenge is the potential for data leakage and privacy violations. Virtual teams depend on digital tools for communication and collaboration, increasing the risk of sensitive information being exposed. Such breaches of privacy can significantly erode trust, as team members may become concerned about the security of their shared data.

To counter these challenges, E-leaders must implement specific strategies to foster trust within virtual teams. One key approach is the use of multi-channel communication, ensuring that information is conveyed clearly and accurately across various platforms. E-leaders should facilitate regular communication that balances formal and informal exchanges, helping to maintain a sense of connection among team members despite physical distance [29]. Additionally, E-leaders should create opportunities for informal interactions, such as virtual “coffee breaks” or informal check-ins [30], which can help foster social bonds and build trust in a relaxed, low-pressure environment. Equally important is the need for E-leaders to establish clear privacy and security measures to protect the team’s sensitive information. By implementing encrypted messaging tools, secure file-sharing platforms, and clear guidelines for data protection, E-leaders can demonstrate a commitment to safeguarding team members’ privacy, reinforcing trust.

#### Task management.

In virtual teams, compared to traditional face-to-face teams, there is a marked increase in diversity in terms of national culture, geographic location, and values [31]. This diversity can significantly influence members’ behaviors, leading to differences in communication styles, decision-making processes, and work ethics [15,20]. For instance, team members from different cultures may have different preferences for how decisions are made. Some may prefer a more collaborative approach, while others may lean toward authoritative decision-making. These differences can complicate task management by introducing misunderstandings or inefficiencies in communication. The adoption of communication tools, such as email, instant messaging, and video conferencing, helps to increase the frequency of communication but also raises the risk of information surplus [32]. Information surplus overwhelms team members, leading to difficulties in prioritizing tasks and making effective decisions [16]. To manage this, E-leaders should implement clear communication protocols, such as filtering essential information and using task-specific communication channels to prevent information surplus and ensure the clarity of key messages. Furthermore, temporal dispersion due to time zone differences reduces the chances for real-time interactions, which poses additional challenges for asynchronous virtual teams [33]. Communication patterns often take the form of multiple monologues rather than interactive dialogues, which can lead to isolation, loneliness, and a diminished sense of mission. The traditional model of face-to-face mutual supervision is impractical in virtual settings, which can lead to some members avoiding their responsibilities. To address this, E-leaders can use task management software to track progress and ensure that all members are held accountable for their tasks.

However, the decentralized nature of virtual teams also provides opportunities for E-leaders to foster motivation and engagement. The sensitivity to social identity cues in such teams enables E-leaders to establish common ground and set clear, shared goals [7]. By considering the unique cultural contexts of team members, E-leaders can guide individuals in setting personalized work goals and adapting their workflows to suit their individual needs. This personalized approach can sustain enthusiasm and help ensure team stability. Additionally, E-leaders play a crucial role in maintaining transparency and accountability. By regularly updating members on task progress through project management tools and maintaining clear action item lists and timelines, E-leaders can ensure that everyone is aligned, and that progress is measurable [34]. Facilitating virtual “check-ins” or collaborative spaces where team members can see each other’s contributions helps foster a collaborative environment and promotes a sense of shared responsibility.

#### Well-being safeguarding.

Virtual teams heavily rely on ICT as the cornerstone of their operations. However, improper usage of ICT can adversely impact employee well-being and, consequently, the effectiveness of virtual teams. Team members in virtual teams are particularly susceptible to stress related to their use of ICT. Whiting & Symon (2020) highlighted the role of “digital housekeepers” who take on various invisible tasks such as managing inboxes, organizing files, and filtering spam [35]. The increased intangible labor required by ICT usage can often overload employees with work, leading to burnout [26]. Moreover, Wepfer et al. (2018) noted that virtual teams tend to blur the boundaries between work and personal life, with team members often responding to work requests outside of their scheduled working hours [36]. This constant connectivity can cause fatigue, stress, and an erosion of personal time, exacerbating the risk of burnout and decreasing overall job satisfaction [32].

Considering these challenges, E-leaders play a crucial role in safeguarding the well-being of team members. Effective E-leaders can manage ICT usage to reduce stress by implementing strategies that help control workload and communication flow. They can introduce clear guidelines to limit after-hours communication, helping to set expectations around work-life balance. For instance, E-leaders can designate “quiet hours” or encourage the use of “do not disturb” modes, ensuring that employees have designated downtime and reducing the constant pressure to be available. E-leaders can also use project management tools to track workloads and ensure that responsibilities are evenly distributed [37]. By regularly monitoring tasks, E-leaders can identify signs of overload early and reassign tasks when necessary [38]. This level of transparency helps prevent digital burnout and promotes a sense of fairness within the team, as no individual is left with an unmanageable workload. By fostering a supportive work environment and utilizing technology to monitor work distribution, E-leaders ensure that employees are not overburdened.

#### Adaptation management.

Virtual teams operate in a decentralized environment and are characterized by high dynamism and flexibility [39]. However, this flexibility presents significant challenges. Virtual teams need to remain sensitive to external changes such as market shifts, technological advances, or changes in organizational priorities, requiring quick and effective adaptation. Without this adaptability, teams risk resource mismatches and can become disconnected from broader organizational goals, diminishing their overall effectiveness [40]. Additionally, unlike traditional face-to-face teams, virtual teams face unique challenges during periods of transformation. The absence of physical communication makes it harder for teams to adapt to technological changes, and the reliance on digital platforms introduces hurdles such as technical difficulties and inadequate training on new tools. The geographic dispersion of team members further complicates coordination, as time zone differences and cultural diversity can hinder alignment on tasks and goals.

To combat the effects of geographical dispersion and reduce negative sentiments, E-leaders can proactively use digital communication tools to facilitate transparency and ensure ongoing dialogue. Regular online meetings via platforms allow for real-time updates, clarifications, and support, helping team members feel more secure and informed about the transformation process. This ensures that the team remains connected and that concerns are addressed promptly. To ensure progress is being made during transitions, E-leaders can rely on real-time monitoring tools to track the status of tasks and ensure alignment with evolving goals [37]. These tools not only provide visibility into the work being done but also allow E-leaders to quickly identify potential issues and intervene before they escalate, making the adaptation process smoother. By using such digital tools to oversee task progress, E-leaders maintain control and visibility in a virtual environment where direct supervision is limited. Finally, E-leaders can evaluate the adaptation process continuously by gathering feedback through survey tools or direct team check-ins [41]. They can offer targeted support such as training on new technologies or facilitate workshops to improve digital collaboration.

#### Innovation management.

Innovation is a crucial driver of team effectiveness, and virtual teams possess distinct advantages in fostering innovation. Due to their geographical dispersion, virtual teams can tap into a diverse pool of talent, skills, and perspectives that may not be available in traditional face-to-face teams. This diversity of thought, coupled with the flexibility of virtual work arrangements, enhances creativity and enables teams to approach problems from multiple angles. Additionally, virtual teams benefit from having access to a wider range of resources, such as cloud-based tools and digital collaboration platforms, that facilitate the exchange of ideas across time zones. ICT plays a vital role in the innovation process, with both synchronous and asynchronous tools supporting different facets of creativity [26]. Synchronous ICT, such as video conferencing tools, allows team members to engage in real-time brainstorming sessions where ideas can be generated, refined, and challenged on the spot. This instant feedback loop encourages dynamic discussions and accelerates the creative process. On the other hand, asynchronous ICT, including collaborative platforms, provides team members with the freedom to explore and develop ideas at their own pace [42]. These tools allow ideas to be stored, edited, and built upon by other team members, fostering a continuous flow of creativity over time.

E-leaders play a critical role in managing the innovation process within virtual teams. They can ensure the appropriate use of ICT by selecting the right tools for different stages of innovation, facilitating both real-time collaboration and independent exploration. Moreover, E-leaders can encourage a culture of open communication, where team members feel comfortable sharing ideas without fear of judgment. They also monitor team dynamics to ensure that all voices are heard and prevent issues such as free-riding or disengagement from hindering the creative process [43]. By using project management tools to track progress, E-leaders help ensure that ideas are translated into actionable tasks, maintaining focus and momentum throughout the innovation cycle.

### Hypothesis development

The above-mentioned dimensions of E-leadership address critical challenges faced by virtual teams. However, their impact on team effectiveness does not stem from any single dimension in isolation but rather from their synergistic interplay. For instance, a leader who fosters trust but lacks task management skills may cultivate a supportive team culture yet fail to ensure efficiency and goal alignment. Conversely, a leader, who excels in task management but neglects adaptation management, may establish structured workflows but hinder the team’s ability to respond flexibly to environmental changes.

Thus, the five dimensions of E-leadership are interdependent within virtual teams. Their collective presence enables leaders to address the multifaceted demands of geographically dispersed and digitally connected work environments. The effectiveness of a virtual team is contingent upon the simultaneous integration of these dimensions, creating a leadership approach that is both structured and adaptable to the complexity of virtual collaboration.

Hypothesis 1: The effectiveness of virtual teams is determined by specific configurations of E-leadership dimensions rather than a single dimension.

Although all five dimensions of E-leadership contribute to virtual team effectiveness, their relative importance varies depending on team structure, industry, and operational context. Different virtual teams face distinct challenges based on their degree of geographical dispersion, cultural diversity, technological dependence, and task complexity. As a result, multiple leadership configurations can lead to equally high effectiveness, as long as the core leadership needs of the team are met.

Highly innovative, flexible teams benefit more from innovation management and adaptation management, as they need to navigate constant technological advancements and knowledge-sharing challenges. Teams operating in high-stress environments may require well-being safeguarding and trust building to reduce burnout and emotional exhaustion while maintaining productivity. Newly formed or culturally diverse virtual teams need stronger trust building and adaptation management to ensure smooth integration and collaboration, as misalignment in work styles and expectations can otherwise lead to conflicts and inefficiencies. These different leadership configurations illustrate that there is no single optimal leadership formula. Instead, virtual teams can achieve effectiveness through distinct pathways, as long as the leadership dimensions align with their structural and operational needs.

Hypothesis 2: Different configurations of E-leadership dimensions are equivalent to improving the effectiveness of virtual teams.

The necessity of each dimension of E-leadership is determined by a dynamic balance between the excess of ability and the short-board effect as virtual teams progress through different levels of performance. At lower performance levels, teams require a foundational set of leadership dimensions that ensure basic operational functionality. At this stage, the demands on leadership are more straightforward, and having leadership dimensions that exceed the immediate needs of the team may be less impactful. In this scenario, an excess of ability may lead to inefficiencies, as the team does not yet face complex challenges that necessitate a broad and sophisticated leadership approach.

However, as the team moves to higher performance levels, the leadership demands become more complex and multifaceted. At these elevated levels, the leadership required to guide the team must not only be broad and adaptable but also maintain a balance across all dimensions. The short-board effect comes into play: if any leadership dimension falls short of meeting the team’s needs, it can undermine the effectiveness of the other dimensions, regardless of their strength. In other words, even if certain leadership dimensions are at an advanced level, one dimension failing to meet its required threshold can result in an overall failure to meet performance expectations. This is because all dimensions of leadership contribute to the team’s overall functionality and success. The team’s ability to adapt to changes, sustain innovation, and maintain cohesion will be compromised if any leadership dimension is neglected, regardless of the others’ effectiveness.

Therefore, while the relative importance of each leadership dimension changes as the team evolves, it is critical that each dimension of E-leadership is maintained at a sufficient level. When the team is aiming for higher performance, the interdependence of E-leadership dimensions means that an imbalance may result in reduced effectiveness. A balanced leadership approach that addresses all dimensions at appropriate levels is essential for sustaining high performance in virtual teams.

Hypothesis 3: Each dimension of E-leadership holds its importance for achieving virtual team effectiveness, with varying degrees of importance across different performance thresholds.

## Methodology

### Research method

fsQCA is a set-theoretic method that allows us to shift our attention from independent variables to holistic configurations [44]. Unlike traditional quantitative methods, which fail to unravel the reciprocal and nonlinear relationships among antecedents leading to an outcome, fsQCA identifies multiple pathways to achieve the same final state. Based on configuration theory, the combination of antecedent variables may have a synergistic effect, with different combinations achieving similar results through various paths [45]. While existing research suggests that different dimensions of E-leadership promote virtual team effectiveness, they do not hold equal importance. The value of E-leadership lies in the correct allocation of “recipes” [46]. fsQCA enables us to analyze a medium number of cases with a relatively large number of causal factors, making it ideal for studying configurations of E-leadership practices, even with a small and unrepresentative sample [47].

NCA identifies the necessary conditions from the aspects of “in degree” for achieving a certain result [48]. Necessary conditions are those that must exist to achieve an outcome, often referred to as the “one-vote veto.” While fsQCA can identify necessary conditions, it cannot determine their degrees of necessity [49]. Bottleneck conditions, as identified through the NCA analysis, refer to threshold-specific constraints that must be addressed to enable virtual teams to progress to higher levels of effectiveness. These conditions act as critical barriers. If not sufficiently developed, they prevent improvements in team performance regardless of the strength of other dimensions. Unlike compensatory factors where strengths in one area can offset weaknesses in another, bottleneck conditions require a minimum threshold to be met for further progress. For example, while high levels of all six E-leadership dimensions are not required, the absence of any dimension may adversely affect virtual team effectiveness [8].

The rationale for combining fsQCA and NCA lies in their ability to address different aspects of the same research question. fsQCA excels at identifying multiple causal configurations that can lead to high virtual team effectiveness. This set-theoretic approach allows us to capture the complex, non-linear relationships among E‑leadership dimensions and their combined impact on team outcomes. On the other hand, NCA identifies the necessary conditions for team effectiveness, shedding light on the minimum thresholds of E‑leadership dimensions required to achieve desired outcomes. While fsQCA provides insight into the “recipes” of E‑leadership configurations, NCA highlights the critical bottleneck conditions that must be addressed for team performance to improve, regardless of the configuration. By combining these two methods, we offer a more nuanced, multi-dimensional analysis that would not be possible using either method alone.

It is important to note that both fsQCA and NCA are fundamentally set-theoretic and deterministic methods; therefore, they do not provide traditional inferential statistics, such as confidence intervals or margins of error for thresholds. In fsQCA, robustness is typically assessed through sensitivity analyses, for example, changing the consistency and frequency thresholds to ensure the stability of configurations. In NCA, statistical inference is supported through permutation tests and ceiling accuracy metrics, but confidence intervals are not a standard component of the analytical framework. As such, while our findings indicate necessary conditions and bottlenecks, these should be interpreted in the context of these methods’ assumptions and limitations, rather than as probabilistic estimates.

### Design of scale

To investigate the proposed model, we invited virtual teams from manufacturing enterprises in Zhejiang Province, China, to participate in the study. Zhejiang Province, located on China’s eastern coast, is one of the country’s most populous and economically vibrant regions. As of 2023, Zhejiang’s population exceeds 66.2 million, and its GDP has surpassed $1.4 trillion USD, making it one of the top contributors to China’s national economy. The province is renowned for its innovation-driven growth, with a strong emphasis on advanced manufacturing, digital transformation, and global trade. Manufacturing enterprises in Zhejiang have increasingly adopted automation and virtual collaboration tools such as DingTalk, enterprise WeChat, and Zoom to maintain their competitive edge in domestic and international markets. These virtual teams exhibit notable variation in structure and function, ranging from decentralized R&D collaborations to centralized remote factory operations and hybrid sales coordination teams. This diversity provides a rich context to explore how different digital tools and team structures influence E-leadership dynamics. The enrolling criteria stipulate that participating virtual teams consist of geographically dispersed members actively collaborating on shared projects. Teams were selected from manufacturing enterprises to ensure consistency in operational complexity, task interdependence, and reliance on digital technology. These factors are highly relevant for studying virtual team dynamics and E-leadership. Moreover, the manufacturing sector in Zhejiang epitomizes the broader trend of digital transformation sweeping across Chinese economy. Compared to many other industries, manufacturing firms in this region are early adopters of smart technologies, hybrid coordination systems, and enterprise-wide digital platforms. These characteristics that make them an ideal empirical setting for studying virtual collaboration.

The survey instrument was developed systematically to ensure reliability and validity. The initial questionnaire was based on existing literature and included dimensions of E-leadership (task management, trust building, adaptation management, well-being safeguarding, and innovation management) and virtual team effectiveness. We adapted the scales from Roman et al. (2019) which provides the scales of task management, trust building and adaptation management [8]. Every dimension has three items. The scales of well-being safeguarding and innovation management are from Chamakiotis et al. (2021) [26]. Virtual team effectiveness was assessed using scales adapted from Alper et al. (2000) [50]. Respondents rated items on a 7-point Likert scale ranging from 1 (not at all true) to 7 (very true). The survey instrument was refined and validated through a pilot test. Two researchers independently translated the English version into Chinese, followed by a discussion to refine the wording for cultural and linguistic appropriateness. To further validate the survey instrument, a pilot study was conducted with 10 virtual teams, involving a total of 83 respondents. Participants provided feedback on clarity, relevance, and appropriateness of the questions, leading to refinements in item phrasing and response scale adjustments.

### Data collection and measurement

The sampling strategy targeted manufacturing enterprises in Zhejiang Province, given their extensive reliance on virtual teams to manage geographically dispersed operations. Data collection was distributed via email and conducted in two phases to minimize method bias. The first phase, in June 2023, focused on assessing E-leadership practices, while the second phase, a month later, measured team effectiveness. This approach reduces common method variance and ensures a more accurate representation of the relationships under investigation. Invitations were sent to 200 teams, with a participation agreement from 117 teams. After data cleaning, 74 complete and valid responses were included in the analysis, resulting in a response rate of 37%. This response rate, while moderate, reflects the challenges of data collection in organizational settings, where access to virtual teams is often limited by confidentiality concerns and operational constraints. Despite these limitations, the sample provides valuable insights into the dynamics of virtual teams in a high-tech and competitive economic environment. In addition, fsQCA and NCA are well-suited to studies with small to medium samples. fsQCA can analyze configurational relationships effectively with fewer cases, as its focus is on causal complexity rather than universal generalizations [47]. Similarly, NCA identifies necessary conditions and their thresholds, providing valuable insights even in small samples [49]. These methods ensure meaningful findings by examining configurational patterns and necessary conditions rather than relying solely on statistical power. Prior fsQCA and NCA studies have drawn meaningful insights with samples as small as 28–59 cases [51–52]. Therefore, the current sample size is sufficient to produce theoretically valuable and methodologically robust conclusions about the effectiveness of E-leadership in virtual teams. Table 2 summarizes the quartile distribution, mean and standard deviation of E-leadership and virtual team effectiveness.

**Table 2:** Descriptive Statistics.

Varibles	Mean	Standard deviation	25th percentile	50th percentile	75th percentile
Trust building	5.49	0.95	4.67	5.67	6.33
Task management	4.45	1.06	4.00	4.33	5.33
Well-being safeguarding	5.03	1.09	4.33	5.00	5.67
Adaptation management	4.81	1.01	4.33	4.67	5.33
Innovation management	4.83	0.95	4.00	4.84	5.59
Virtual team effectiveness	4.86	0.71	4.47	4.60	5.53

To evaluate the reliability and validity of the measurement model, partial least squares structural equation modeling was used. Reliability was assessed through Cronbach’s alpha coefficients and composite reliability scores, which exceeded the 0.7 threshold for all variables [53]. Validity was confirmed using average variance extracted (AVE) scores, all of which were above 0.5. Model fit was evaluated using the standardized root mean square residual (SRMR), which met the criterion of ≤ 0.08 with a value of 0.072. These results indicate that the measurement scales are reliable and valid for the study.

## Sufficiency analysis based on fsQCA

### Data analysis

fsQCA encompasses three primary phases: data calibration, necessity analysis of individual conditions, and configuration analysis. Our initial step involves calibrating the data. Both the causal conditions and the outcome are represented by fuzzy scores, which are based on indirect calibration or direct calibration, ranging from 0.00 for full non-membership to 1.00 for full membership [44]. Considering our data is derived from a Likert scale, a direct calibration was deemed more suitable. We find that the values of trust building, well-being safeguarding, and innovation management are generally high and do not conform to common distribution patterns. This is most likely because these variables represent internal relationships and important capabilities within the team, so participants overestimated them. This affects the accuracy of fsQCA results. Therefore, we use different calibration standards to calibrate those variables. Thresholds were set at 6 for full membership and 2 for complete non-membership for adaptation management, task management and virtual team effectiveness. Thresholds were set at 6.5 for full membership and 3 for complete non-membership for trust building, well-being safeguarding, innovation management. After calibration, a necessity analysis of individual conditions was performed. The results reveal no antecedent exceeds the conventional 0.9 threshold, which means no singular dimension of E-leadership can be considered necessary in the set of cases under scrutiny to render virtual team effectiveness. Accordingly, we delve into a more detailed discussion on the configurations of E-leadership.

As usually recommended, a frequency threshold of 1 was applied, meaning each configuration had to be supported by at least one observed case to be considered. This threshold is appropriate for medium-sized samples, ensuring that all meaningful configurations are included while excluding rare or spurious ones. A consistency threshold of 0.8 was used to determine whether a configuration reliably leads to high virtual team effectiveness. This threshold reflects the proportion of cases in which a configuration is associated with the outcome and is commonly used in fsQCA studies [47]. To address causal asymmetry, a PRI threshold of 0.8 was applied. This ensured that configurations contributing to the presence of the outcome did not simultaneously lead to its absence.

**Fig 1:**
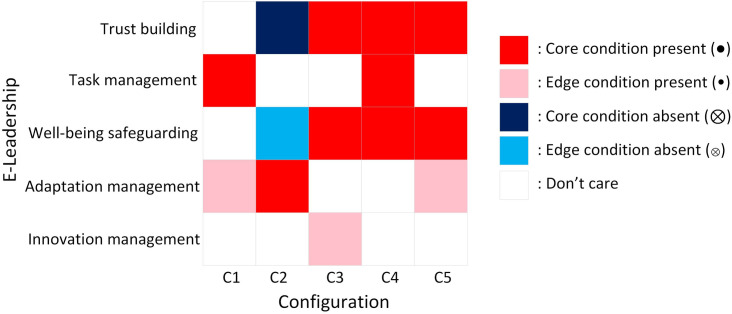
Heatmap of conditions across fsQCA configurations.

The outcomes of fsQCA comprised three solutions: complex solution, intermediate solution, and parsimonious solution. The complex solution includes all observed configurations without minimization, while the parsimonious solution simplifies configurations extensively by including all logical remainders. The intermediate solution, selected for interpretation, balances complexity and parsimony by incorporating theoretically informed simplifying assumptions [54]. We combined the results of intermediate solution and parsimonious solution, regarding intermediate solution as the edge condition and parsimonious solution as the core condition. Configurations consist both core conditions and edge conditions. Among these, core conditions are of paramount importance within the configuration, as they serve as the primary drivers of the outcome. Core conditions are the central elements that must be present for the configuration to result in the desired outcome. Edge conditions play a supportive or supplementary role. The analysis of configurations typically places greater emphasis on core conditions, as they are the critical determinants of success or failure in the given configuration. Five configurations of E-leadership were revealed that potentially lead to high virtual team effectiveness. As shown in Table 3, the consistency (CS) of all configurations is greater than 0.8, indicating all configurations satisfy the consistency condition [54]. An overall consistency (OCS) of 0.932 and an overall coverage (OCV) of 0.894 guarantee reliable interpretation of the results. To enhance the interpretability of the fsQCA results, a heat map (Fig 1) has been added to visually illustrate the strength and presence of each E-leadership dimension across the five configurations. Darker shades indicate core conditions, while lighter shades represent peripheral conditions, allowing for a more intuitive comparison of configuration patterns.

**Table 3:** Configurations of fsQCA.

	C1	C2	C3	C4	C5
Trust building		**⊗**	●	●	●
Task management	●			●	
Well-being safeguarding		⊗	●	●	●
Adaptation management	•	●			•
Innovation management			•		
Consistency	0.971	0.983	0.978	0.984	0.965
Raw coverage	0.698	0.311	0.416	0.519	0.506
Unique coverage	0.143	0.040	0.007	0.035	0.022
Solution consistency			0.943		
Solution coverage			0.844		

Note: Black circles (●) denote the existence of the condition; circles with “x” (**⊗**) denotes the absence of the condition. Large circles denote the core condition; small circles denote the edge condition. Blanks denote the “don’t care” condition in the configuration.

The research findings reveal five configurations of E-leadership that contribute to high virtual team effectiveness. This result highlights that a single leadership capability alone cannot ensure high team performance. On the contrary, it is the specific combination of E-leadership dimensions that serves as a sufficient condition for achieving high virtual effectiveness, thus supporting the notion that the configuration, or “formula,” is more critical than the individual components [46]. Each configuration is tailored to the unique characteristics of the team and its external environment, offering an understanding of how leadership practices must adapt to different circumstances.

Configuration C1, with its highest unique coverage, represents the most representative virtual team, suggesting that task management is the core condition, supported by adaptation management. This configuration is particularly suited to virtual teams operating in highly complex environments, where clear task structuring and the ability to adapt to changing circumstances are critical. These teams are likely to face external challenges such as rapidly shifting market conditions or technological disruptions, which require E-leaders to focus on both operational efficiency and continuous adaptation. The emphasis on task management helps mitigate the risks of team members experiencing overload or isolation, while adaptation management ensures the team’s capacity to respond flexibly to environmental changes, fostering a collaborative and resilient environment.

In configuration C2, the core conditions include a lack of trust building and adaptation management. This configuration corresponds to virtual teams operating in unstable, high-pressure environments, where survival depends on continual adaptation and short-term performance. E-leaders are expected to focus on delivering results under dynamic and uncertain conditions, which may come at the cost of long-term team cohesion and trust. The absence of trust building and well-being safeguarding mechanisms can lead to higher member turnover and reduced team stability. While this mode may yield short-term effectiveness, it is unsustainable in the long run, especially as external conditions stabilize or as task complexity increases over time.

Configurations C3, C4, and C5 all share the core conditions of trust building and well-being safeguarding, which are essential for ensuring team stability and long-term effectiveness. These configurations are the most suitable for teams that require ongoing collaboration and strong interpersonal relationships. The establishment of trust promotes open communication and fosters collaboration over long distances, while well-being safeguarding ensures that team members remain engaged and mentally healthy. Beyond these foundational elements, each of these configurations focuses on additional leadership dimensions suited to the team’s specific needs. Configuration C3 emphasizes innovation, highlighting the importance of creativity and problem-solving, especially in industries that require continuous innovation. Configuration C4 prioritizes task completion, addressing the needs of geographically dispersed teams facing complex tasks that demand structured workflows. Finally, configuration C5 places a stronger focus on adaptation management, emphasizing flexibility and agility. This is particularly relevant for teams operating in industries that are subject to frequent and unpredictable changes, where continuous adaptation to new circumstances is a vital condition for survival.

The variations across these configurations suggest that virtual teams are not monolithic entities. They are diverse in terms of their developmental stages, external challenges, and operational requirements. E-leaders must be capable of tailoring their leadership strategies to these specific characteristics, adjusting their focus on different E-leadership dimensions based on the team’s context and needs.

### Robustness of fsQCA analysis results

In fsQCA, results are considered robust if slightly different raw data processing methods lead to similar consistency, coverage and configuration results [55]. Robustness tests ensure that the findings are not artifacts of methodological choices or parameter settings. To validate our results, we conducted several robustness checks by systematically varying key thresholds: the PRI threshold, the raw consistency threshold, and the calibration thresholds for full membership and non-membership.

First, we varied PRI threshold from 0.8 to 0.78. This test assesses the extent to which configurations remain consistent without contributing to contradictory outcomes. As shown in Table 4, the identified configurations remained largely consistent with the original analysis, except for configuration C3. The absence of innovation management act as a core condition in C3. This configuration suggests that the corresponding virtual team functions in a highly mature industry, diminishing the necessity for innovation. In fact, engaging in innovation activities could potentially elevate the operational expenses of the virtual team, consequently detracting from the team’s operational efficiency. This sensitivity underscores the contextual dependence of certain conditions.

**Table 4:** Robustness tests of fsQCA.

		C1	C2	C3	C4	C5
change PRI threshold from 0.8 to 0.78	Trust building		⊗	●	●	●
	Task management	•				•
	Well-being safeguarding		⊗		•	•
	Adaptation management	●	●	●		
	Innovation management			⊗	•	
	Consistency	0.971	0.983	0.932	0.978	0.984
	Raw coverage	0.698	0.311	0.468	0.416	0.519
	Unique coverage	0.100	0.034	0.044	0.018	0.035
	Solution consistency	0.930				
	Solution coverage	0.866				
change raw consistency threshold from 0.8 to 0.9	Trust building		**⊗**	●	●	●
	Task management	●			●	
	Well-being safeguarding		⊗	●	●	●
	Adaptation management	•	●			•
	Innovation management			•		
	Consistency	0.971	0.983	0.978	0.984	0.965
	Raw coverage	0.698	0.311	0.416	0.519	0.506
	Unique coverage	0.143	0.040	0.007	0.035	0.022
	Solution consistency	0.943				
	Solution coverage	0.844				
change thresholds for full membership from 6 or 6.5 to 5.5 and 2 or 3 to 1.5	Trust building		**⊗**	•		•
	Task management	●		●		
	Well-being safeguarding		⊗	•		●
	Adaptation management	•	●			●
	Innovation management					
	Consistency	0.971	0.971	0.982		0.954
	Raw coverage	0.698	0.258	0.499		0.480
	Unique coverage	0.155	0.043	0.062		0.042
	Solution consistency	0.946				
	Solution coverage	0.856				

The raw consistency threshold, which measures how reliably a configuration leads to the outcome, was varied from 0.8 to 0.9. The increase in the consistency threshold did not significantly alter the identified configurations or their associated coverage values. This stability indicates that the configurations exhibit strong causal relationships with the outcome and are not dependent on marginal cases. The robustness of the configurations across different consistency thresholds strengthens the validity of the findings.

Calibration thresholds for fuzzy set membership were adjusted to test their impact on the results. We change the thresholds for full membership and full non-membership from 6 or 6.5 to 5.5 and 2 or 3 to 1.5. This adjustment ensures that the calibration process captures sufficient variability in the data while avoiding overfitting. Despite these changes, the overall consistency and coverage of the configurations remained stable. The results demonstrate that the model is not overly sensitive to minor variations in calibration thresholds, confirming its robustness.

The consistency of results across different thresholds implies that the identified configurations of E-leadership dimensions are robust and reliable. The variation in PRI threshold highlights the importance of causal asymmetry in fsQCA. The emergence of C3 as sensitive to the PRI threshold indicates that innovation management’ s role is context-dependent, particularly in industries with high operational maturity. Stability across different raw consistency thresholds suggests that the configurations are strongly linked to the outcome, reducing concerns about spurious relationships. The insensitivity of configurations to calibration threshold changes demonstrates that the fuzzy set memberships were well-calibrated, providing confidence in the data transformation process. These robustness tests collectively validate the model’s ability to identify meaningful and stable configurations.

## Necessity analysis based on NCA

### Data analysis

While the findings from the fsQCA analysis indicate that a singular dimension of E-leadership does not necessarily influence virtual team effectiveness, this conclusion is derived purely from a qualitative standpoint. We posit that the necessity “in degrees” of E-leadership dimensions may be reflected in different levels of virtual team effectiveness. To delve deeper into the influence of E-leadership on virtual team effectiveness, we employ NCA for further investigation by applying the NCA package in R. The steps of analysis include drawing ceiling lines, calculating ceiling parameter accuracy, ceiling zone, and scope, and drawing the bottleneck table [56]. We use two upper bound techniques to obtain envelopes and calculate the relevant parameters. It is important to note that the performance levels presented in the bottleneck table are derived from a continuous percentage scale (0–100), where values reflect normalized performance rankings. Therefore, each level corresponds to a relative position in the outcome distribution rather than an absolute frequency count, and the number of teams is approximately evenly distributed across performance levels. The results demonstrate that all five dimensions of E-leadership function as necessary conditions to varying degrees. These necessity relationships become particularly evident through the bottleneck analysis, which reveals how the threshold requirements for each dimension change depending on the desired level of team performance. This highlights that E-leadership influences virtual team outcomes in a gradual and context-dependent manner.

### Findings

**Fig 2:**
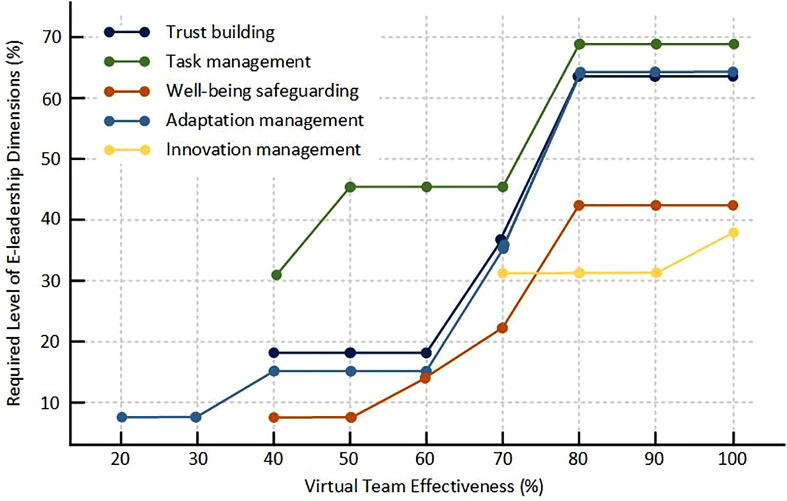
Required level of E-leadership dimensions.

The bottleneck table (Table 5) shows the varying degrees of necessity of E-leadership dimensions required to achieve different levels of virtual team effectiveness. We classify the levels of virtual team effectiveness as follows: 0%−50% as low effectiveness, 51%−80% as medium effectiveness, and 81%−100% as high effectiveness. To further illustrate these findings, we added a visual summary (see Fig 2), which depicts the threshold levels of each E-leadership dimension required across different levels of team effectiveness. The results suggest that different levels of virtual team effectiveness require varying levels of E-leadership dimensions, which highlights the evolving needs of virtual teams as they mature.

**Table 5:** Bottleneck results of NCA.

Virtual team effectiveness	Trust building	Task management	Well-being safeguarding	Adaptation management	Innovation management
0	NN	NN	NN	NN	NN
10	NN	NN	NN	NN	NN
20	NN	NN	NN	7.3	NN
30	NN	NN	NN	7.3	NN
40	18.3	30.7	7.3	14.3	NN
50	18.3	46.2	7.3	14.3	NN
60	18.3	46.2	14.3	14.3	NN
70	36.5	46.2	21.4	35.8	30.7
80	63.8	69.3	42.8	64.2	30.7
90	63.8	69.3	42.8	64.2	30.7
100	63.8	69.3	42.8	64.2	38.3

Note: Performance levels are based on normalized rankings of team effectiveness (0–100 scale), with evenly distributed case counts across the spectrum.

At low effectiveness levels (0%−50%), teams primarily need to focus on task management. This dimension helps establish a foundation for team collaboration, ensuring that basic operational goals and roles are clear. Task management at this stage ensures that teams have clear objectives, defined roles, and streamlined processes, which are essential for stabilizing the team and creating an environment where further leadership practices can take root. Without solid task management, teams may struggle with coordination, making it difficult to move beyond the initial stages of collaboration.

As teams progress toward medium effectiveness levels (51%−80%), the need for trust building, adaptation management, and well-being safeguarding grows. Trust building becomes crucial as the team expands its interactions and starts facing more complex tasks. Communication often happens through technology rather than face-to-face interactions. Without trust, team members may hesitate to collaborate fully or share ideas openly, limiting the team’s potential. Along with trust building, adaptation management rises in importance as teams face the need to respond to changes, both internal and external. In dynamic environments, virtual teams must be flexible and adaptable to evolving situations, whether it’s technological advancements, market shifts, or internal restructuring. Adaptation management equips teams with the ability to handle change proactively. Furthermore, well-being safeguarding becomes a critical factor as teams start handling more tasks and challenges. Virtual teams, particularly in high-stress environments, may experience burnout, stress, and fatigue, which can hinder performance. Leaders must prioritize the mental health and well-being of their team members to ensure sustained productivity and morale.

For teams striving to achieve high effectiveness (81%−100%), all dimensions of E-leadership become necessary, with varying levels. At this stage, teams need a balance among all the leadership dimensions to function effectively. Innovation management takes on a particularly pivotal role at higher effectiveness levels because teams operating at this stage are often tasked with solving complex, novel problems and driving continuous improvements. Innovation management fosters creativity, idea generation, and the ability to think outside the box, which are essential for teams to stay competitive and push boundaries in their work. Without innovation management, teams risk stagnation, and their problem-solving capabilities may become limited, particularly in industries requiring constant change and improvement.

We further find that there are some bottleneck conditions at some level of effectiveness. When there is a bottleneck, raising the level of any other conditions is ineffective. The actual level of a condition exceeding the threshold level at a given effectiveness level is called conditional inefficiency [48,56]. Therefore, when a virtual team is seeking to achieve higher effectiveness, the level of the condition creating the bottleneck must be improved to the threshold required for an expected outcome.

Well-being safeguarding is a bottleneck condition at the interface between low and medium effectiveness levels. Teams that fail to address the well-being of their members will struggle to progress beyond a low level of effectiveness. If well-being concerns such as burnout, stress, and emotional disengagement are not addressed, even high-quality task management or trust-building will be ineffective at improving team performance. In teams at the initial stages of development or those struggling to meet performance goals, focusing on the well-being of team members is critical. Leaders should implement strategies to promote mental health, reduce stress, and foster a supportive team environment. Team-building activities, mental health support, flexible schedules, and wellness programs can be instrumental in improving the foundational health of the team, enabling it to progress to higher effectiveness levels.

Adaptation management is critical for teams aiming to increase their effectiveness from medium to high levels. If teams are unable to adapt to external changes such as shifts in market dynamics, technological advancements, or internal challenges, they will face a bottleneck that restricts further growth. Leaders should ensure that teams are equipped with tools and knowledge to make rapid adjustments in the face of change. Training in adaptive leadership and fostering an environment where feedback loops are prioritized will enable teams to stay ahead of disruptions. This will allow virtual teams to maintain and enhance their effectiveness even as external factors evolve.

Innovation management becomes a bottleneck condition when virtual teams aim to high effectiveness, as illustrated in Table 4, this dimension necessity emerges at 70% virtual team effectiveness. At medium effectiveness levels, teams may rely on existing strategies, but as they move toward high effectiveness, innovation becomes crucial for sustaining growth and performance. Without a focus on innovation, teams risk stagnation and failure to adapt to changing environments. Leaders must focus on encouraging creativity and new ideas as teams transition to higher levels of effectiveness. Teams should be encouraged to experiment, innovate, and constantly rethink existing processes. This can be achieved by creating a culture that rewards risk-taking, offers creative challenges, and allows for continuous feedback. Teams that do not innovate will struggle to reach 100% effectiveness, limiting their ability to sustain high performance.

To illustrate, we test sample 16, with 34.69% for virtual team effectiveness and 27.27%, 23.08%, 35.71%, 71.43%, and 92.31% for trust building, well-being safeguarding, adaptation management and innovation management, respectively. For sample 16 to increase virtual team effectiveness to the 40% level, the level of task management (23.08%) at least needs to be at 30.7%, and the levels of the other four E-leadership dimensions are already way beyond the required thresholds which are 18.3%, 7.3%, 14.3%, NN for trust building, task management, well-being safeguarding, adaptation management and innovation management, respectively. Here comes the conditional inefficiency for these four E-leadership dimensions at 40% effectiveness level. Therefore, when E-leaders want to reach a targeted effectiveness level, it is advised to save resources from inefficient conditions for the bottleneck condition: directing more attention to improving task management and less attention to other E-leadership dimensions is a rational strategy to fully utilize the limited team resources.

## Discussion

This study aims to explore how different E-leadership practices affect the effectiveness of virtual teams. The analysis, employing fsQCA and NCA, addressed two research questions: (1) How do different configurations of E-leadership functions influence the effectiveness of virtual teams? (2) What are the necessary conditions for high-performing virtual teams in the context of E-leadership? Our findings show that virtual team effectiveness is determined by the integration of multiple E-leadership dimensions, rather than any one function. This supports the argument that a holistic approach to leadership is crucial for managing virtual teams successfully. We found that E-leadership functions do not have a singular, independent effect on virtual team effectiveness; instead, it is the configuration of these leadership dimensions that contributes significantly to team success.

### The combined use of fsQCA and NCA

To gain deeper insights, we apply fsQCA and NCA jointly. Interestingly, while the two methods are largely divergent, they also provide complementary perspectives. fsQCA reveals multiple effective configurations of E-leadership dimensions and demonstrate how different combinations jointly lead to equifinal team effectiveness. In contrast, NCA identify individual necessary conditions that must be present, to a certain degree, for effectiveness to occur. For instance, trust building and task management emerge as necessary across different performance levels in NCA but did not always appear in every fsQCA configuration. This difference is not contradictory but rather complementary. Here are two situations where attributes appear in fsQCA but not in NCA: 1. Configuration-specific relevance: In fsQCA, an attribute may appear in one of several effective configurations leading to virtual team effectiveness. However, since it is not present across all virtual team effectiveness configurations, NCA does not consider it a necessary condition. 2. Substitutable conditions: fsQCA may show that some attributes can each be part of different successful configurations. Since either can substitute for the other, neither is always required, so NCA excludes both as necessary, even though they are important in fsQCA. Therefore, an attribute may contribute significantly in fsQCA without being identified in NCA, highlighting how the two methods offer complementary but distinct insights into causal relationships.

The combined use of fsQCA and NCA generated a more nuanced understanding than that would have been possible through traditional statistical techniques like regression analysis. Regression assumes linear, net effects and emphasizes average tendencies, whereas fsQCA captures causal asymmetry and configurational complexity, and NCA uncovers bottlenecks and boundary conditions that must be met for any success to occur. By using both methods, we were able to cross-validate key insights: NCA confirm the criticality of certain functions identified in fsQCA, while fsQCA uncover multiple successful pathways that NCA alone could not reveal. Here is a comparative summary of how fsQCA and NCA analyze the influence of different E-leadership dimensions on virtual team effectiveness (Table 6).

**Table 6:** Summary of fsQCA and NCA.

E-leadership Dimension	fsQCA Core/Edge Role	NCA Necessity & Bottleneck
Trust Building	Core in C2, C3, C4, C5	Necessary at medium/high levels; not a bottleneck
Task Management	Core in C1, C4	Necessary at all levels; not a bottleneck
Well-being Safeguarding	Core in C3, C4, C5; edge in C2	Necessary at medium/high levels; not a bottleneck
Adaptation Management	Core in C2, edge in C1, C5	Necessary at medium/high levels; Bottleneck from medium to high effectiveness
Innovation Management	Edge in C3	Necessary at high levels; Bottleneck for achieving high effectiveness

The methodological combination of fsQCA and NCA offers significant potential for application in other virtual leadership research contexts. For example, these methods can be employed to examine leadership effectiveness in hybrid teams, cross-cultural virtual collaborations, or crisis-driven remote work environments. fsQCA is particularly suitable for identifying diverse configurations of leadership behaviors that are effective under different structural or cultural conditions, revealing how context shapes leadership outcomes. NCA, on the other hand, uncover threshold levels of critical leadership dimensions such as digital communication skills, emotional intelligence, or cultural sensitivity that are necessary for specific performance outcomes. Together, the methods can help scholars explore both the variety of effective leadership approaches and the indispensable conditions that must not be absent, thus offering a more comprehensive understanding of how leaders enable virtual team success across various organizations under different situations.

### Verification of hypothesis

Hypothesis 1 was strongly supported. Results in Table 4 show that different E-leadership attributes are required to promote virtual team effectiveness at the same time, a single dimension is insufficient. This reflects the growing recognition in leadership theory that virtual teams require more than just a single leadership dimension; instead, they benefit from the synergistic effect of multiple integrated practice. Hypothesis 2 was also confirmed, results in Table 4 present that E-leadership dimensions working together in 5 equifinal ways to promote virtual team effectiveness. This suggests that e-leaders should be flexible, selecting practices that suit their team’s specific situations rather than adhering to a rigid approach. Hypothesis 3 was also supported in our findings, which indicate that as virtual teams mature, there is an increasing demand for more sophisticated and dynamic E-leadership practices. This trend reflects the evolving complexity of virtual team operations, where effective leadership must continuously adapt to emerging challenges and foster an environment that promotes both flexibility and creative problem-solving. In essence, our results underscore the necessity for E-leadership to evolve in tandem with team development, ensuring that leadership practices remain aligned with the changing needs of high-performing virtual teams. Table 7 summarizes the research questions, findings, and implications.

**Table 7:** Research questions, findings and implications.

Research Questions	Corresponding Hypothesis	Key Findings	Practical Implications
What combinations of E-leadership dimensions promote virtual team effectiveness?	Hypothesis 1 Hypothesis 2	Virtual team effectiveness is determined by the configurations of multiple E-leadership dimensions rather than by any single ones. Multiple configurations achieve equifinal results.	E-leaders should adopt an integrated and flexible E-leadership approach. They can tailor leadership practices to their team’s specific strengths and needs.
What are the necessary E-leadership attributes under varying degrees of virtual teams performance?	Hypothesis 3	Each E-leadership dimension plays a necessary role at different stages of team development, with varying importance across different performance levels.	E-leaders should adjust their practices as teams evolve, focusing on foundational coordination in early stages and emphasizing innovation and adaptability as teams mature.

The mechanism behind these results is rooted in the unique dynamics of virtual teams. Virtual teams consist of members who are geographically dispersed and rely heavily on digital technology for all aspects of communication and collaboration. This environment inherently challenges traditional forms of leadership by reducing opportunities for face-to-face interaction, which can lead to information asymmetry and coordination difficulties. In such contexts, effective E‑leadership is essential—not only to establish a stable operational foundation but also to foster a culture of adaptability and continuous innovation. The role of the E‑leader is not to master every dimension of E‑leadership simultaneously. Instead, effective E‑leadership involves strategically identifying and prioritizing those leadership functions that are most relevant to the team’s specific context and challenges. Our configurational analysis demonstrates that different combinations of E‑leadership practices are optimal for different types of virtual teams, depending on factors such as operational complexity and external environmental pressures. This highlights that the overall effectiveness of E‑leadership lies in its adaptive and context-sensitive application, ensuring that critical bottlenecks are addressed so that the team can achieve and sustain high performance. As virtual teams evolve, the E-leadership demands change. In the early stages, a strong emphasis on fundamental coordination practices is vital to establish clear roles and streamline processes, thereby enabling the team to stabilize. As teams progress, additional leadership functions such as building trust, facilitating adaptation, and ensuring member well-being become increasingly critical for navigating more complex and dynamic challenges. At the highest levels of effectiveness, the capacity for innovation is paramount, allowing teams to maintain competitive performance through creative problem-solving and continuous improvement.

While this study focuses on virtual teams within manufacturing enterprises in Zhejiang Province, the core challenges it addresses including trust-building, communication efficiency, coordination, and motivation are widely recognized across industries that rely on virtual collaboration. For instance, in the healthcare sector, cross-functional virtual teams, such as telemedicine units and administrative coordination groups, often face challenges related to information-sharing accuracy, role clarity, and trust between clinicians and support staff. Similarly, in the financial services industry, virtual teams managing international portfolios and risk analysis projects frequently experience communication delays, facing difficulties in aligning strategic priorities, and barriers to cultivating team cohesion. In the education sector, virtual academic teams encounter comparable issues related to maintaining engagement and alignment in remote settings. All those situations suggest that, although the manufacturing context in Zhejiang provides a distinct empirical setting, the mechanisms of E-leadership identified here reflect broader patterns observed in virtual work environments.

### Limitations and future research

The current study presents some limitations. One key limitation is that all participating virtual teams were drawn from manufacturing enterprises in Zhejiang Province, China. This industry- and region-specific sample may introduce biases due to the unique socio-economic and cultural context, limiting the broader applicability of the findings. The challenges faced by manufacturing teams may differ from those in other sectors, especially regarding team structures, technological infrastructures, and cultural diversity. Future studies should expand to virtual teams across various industries and geographic regions to improve external validity. In particularly, cross-culture comparisons could reveal whether E-leadership principles are universally applicable or shaped by regional norms, providing more globally relevant insights.

The limited diversity in team composition and contextual data further restricts the study’s explanatory power. We did not collect detailed demographic information such as age, gender, education, or prior virtual work experience, nor did we fully account for variations in the digital tools used across teams. These contextual variables may influence how E-leadership is enacted or perceived. Future research should gather richer demographic and organizational data to better understand how E-leadership practices interact with specific team characteristics. Enrolling teams from diverse backgrounds and industries could also support the development of more context-sensitive leadership models.

Another limitation lies in the absence of in-depth case studies, which makes it difficult to explore how E-leadership operates in real organizational settings. While our quantitative results provide valuable patterns, they lack the depth to capture dynamic leadership processes over time. Future research should incorporate longitudinal case studies to observe how E-leadership behaviors and their impact evolve across different stages of team development. This approach would offer temporal and contextual insights, enhancing both theoretical understanding and practical relevance.

The study also relies on self-reported data to assess both E-leadership and virtual team effectiveness. Despite using a two-phase data collection to reduce bias, self-reporting remains susceptible to social desirability and common method variance. Future studies should consider using multi-source data, including peer or supervisor assessments and objective performance metrics. In addition, AI-driven behavioral analysis, such as natural language processing of digital communications, could uncover leadership behaviors and patterns that traditional surveys might miss.

Finally, while our application of NCA has provided important insights into the threshold effects of various E-leadership dimensions, the methodological constraints of NCA remain a limitation. As a relatively novel analytical technique, NCA’s statistical properties and causal inference capabilities are still being refined. Further research is needed to advance the methodological rigor of NCA, particularly in aspects such as ceiling line determination, and the delineation of bottleneck conditions, to more comprehensively capture the complex dynamics of E-leadership in virtual teams.

### Theoretical contributions

Prior research in E‑leadership has largely focused on developing multidimensional measurement schemes and exploring individual leadership dimensions within digital contexts [5,19,20]. Such studies have provided a foundation for understanding how ICT and leadership intersect, yet they have tended to examine leadership functions in isolation. This approach has left a critical gap in our understanding of the mechanisms by which various E‑leadership dimensions interact to influence organizational outcomes. Moreover, while some scholars have acknowledged the challenges posed by digital transformation, the literature has not sufficiently addressed the contingent nature of E‑leadership. Specifically, how different leadership dimensions vary in their necessity depending on contextual factors such as team maturity and external environmental pressures [24].

Our research addresses these deficiencies by employing a configurational perspective, using methods such as fsQCA and NCA, to demonstrate that the effectiveness of E‑leadership is driven by the synergistic integration of multiple dimensions rather than by any single function. Our findings reveal that while dimensions like task management and trust building serve as foundational prerequisites, other functions such as adaptation management and innovation management become critical only at advanced stages of team development. This dynamic framework not only fills a notable gap in the existing literature but also offers practical insights for E‑leaders. It suggests that rather than striving to master all dimensions equally, effective E‑leaders should strategically focus on those capabilities that are most pertinent to their specific operational challenges and contextual needs. In doing so, our study extends traditional leadership theories by highlighting the contingency and integration of E‑leadership practices in digital environments.

The literature on virtual teams has extensively documented their potential benefits, such as enhanced flexibility and access to diverse talent, as well as the significant challenges they face including communication breakdowns, diminished trust, and cultural disparities [1,2,15]. Although numerous studies have addressed these challenges, much of the existing research has examined virtual team dynamics using linear models or by isolating individual variables, thereby failing to capture the complex, interdependent nature of virtual team interactions. Additionally, the rapidly evolving digital landscape and the increasing reliance on ICT for communication and collaboration call for a more comprehensive approach to understanding virtual team performance.

Our study contributes to virtual team research by integrating the role of E‑leadership as a mediator of human–technology interaction within virtual teams. Through a configurational analysis, we demonstrate that virtual team effectiveness is contingent upon specific combinations of E‑leadership dimensions that align with the unique operational characteristics and external environments of these teams. Our findings indicate that different configurations of E‑leadership can yield similar levels of effectiveness, highlighting that virtual teams are not monolithic; instead, they vary in terms of their developmental stages, external challenges, and operational needs. This framework provides a more nuanced understanding of how virtual teams can achieve high performance through tailored leadership strategies, thus bridging a critical gap in the literature. By emphasizing that effective virtual team management depends on aligning leadership practices with specific team contexts, our research offers actionable insights for practitioners seeking to optimize team dynamics in diverse and dynamic digital environments.

### Managerial implications

This study offers several practical insights for managers seeking to enhance the effectiveness of virtual teams. First, managers should cultivate E-leadership capabilities that emphasize human-technology interaction, including digital communication skills, remote collaboration tools, and virtual presence. As virtual teams differ from traditional ones, leaders should be equipped to manage both online and hybrid workforces. Second, rather than evenly distributing efforts across all leadership dimensions, managers are advised to identify and address the specific bottleneck that most constrains team performance. Our integration of fsQCA and NCA methods highlights how different types of virtual teams face different critical conditions. For example, teams operating in high-pressure or fast-changing contexts require strong adaptation management to move from medium to high effectiveness, while collaboration-intensive teams benefit most from innovation leadership to maintain high performance. Third, effective E-leadership should be dynamic. The leadership requirement of a virtual team evolves with changes in its environment, structure, and development stage. Therefore, managers should regularly reassess their leadership strategies and adjust them accordingly. To guide implementation, Table 8 summarizes five virtual team archetypes along with the key bottlenecks and corresponding leadership strategies. This practical framework allows organizations to match team types with tailored leadership behaviors and to ensure more efficient utilization of the limited management resources.

**Table 8:** Leadership Behavior integrated from fsQCA & NCA.

Virtual Team Type	Contextual Characteristics	Key Bottleneck Condition	Recommended Leadership Behaviors
Highly complex, fast-changing environments (C1)	Teams in highly complex and fast-changing environments, where efficient task execution and rapid response are critical.	Adaptation Management (Key for transitioning from medium to high effectiveness)	Leaders should establish structured workflows while simultaneously enhancing adaptability through agile response training, real-time scenario planning, and dynamic monitoring systems.
High-pressure, unstable environments (C2)	Teams operating in unstable, high-pressure contexts that prioritize immediate, short-term performance over long-term cohesion.	Adaptation Management (Key for transitioning from medium to high effectiveness)	Leaders should implement continuous learning programs and crisis-response protocols, while gradually integrating trust-building measures to stabilize long-term performance.
Collaboration-intensive teams (C3)	Teams that depend on strong interpersonal collaboration and creativity to drive innovation and sustain engagement.	Innovation Management (Key for sustaining high effectiveness)	Leaders should foster a culture of innovation by encouraging cross-functional collaboration, organizing targeted innovation workshops, and establishing incentive schemes to reward creative problem-solving.
Process-heavy, structured teams (C4)	Teams that rely on clear role definitions and operational structure alongside strong interpersonal cohesion.	Well-being Safeguarding (Key for transitioning from low to medium effectiveness)	Leaders should promote mental health and prevent burnout by offering flexible work arrangements, implementing wellness programs, and fostering a supportive culture, while also ensuring clear task allocation and structured collaboration.
Highly adaptive teams (C5)	Teams that require high flexibility and resilience to navigate frequent external changes while maintaining internal trust.	Adaptation Management (Key for transitioning from medium to high effectiveness)	Leaders should foster continuous learning and agility by implementing adaptive methodologies, institutionalizing iterative feedback loops, and embedding proactive change management practices.
